# Effect of Yogurt Intake Frequency on Blood Pressure: A Cross-Sectional Study

**DOI:** 10.1155/2024/8040917

**Published:** 2024-05-03

**Authors:** Xinqi Li, Zhuo Zhao, Lin Na, Wenjing Cui, Xiaona Che, Jing Chang, Xin Xue

**Affiliations:** ^1^Department of Cardiology, The Second Hospital of Jilin University, Changchun City 130000, China; ^2^Department of Cardiology, Xi'an International Medical Center Hospital, Xi'an 710000, China; ^3^Clinical Laboratory, The Second Hospital of Jilin University, Changchun City 130000, China

## Abstract

Yogurt consumption is a significant factor in reducing the risk of hypertension and preventing cardiovascular diseases. Although increasing evidence has emerged regarding the potential benefits of probiotics in hypertension, there is a lack of large, cross-sectional studies assessing the association between yogurt intake and blood pressure parameters. We aimed to evaluate the association between yogurt intake frequency and blood pressure. A cross-sectional study was designed using data from the National Health and Nutrition Examination Survey from 2003 to 2004 and 2005 to 2006. We included 3, 068 adults with blood pressure data and yogurt intake data. Multivariate regression analyses revealed significant inverse associations between yogurt and systolic blood pressure (*P* < 0.05), diastolic blood pressure (*P* < 0.05), and mean arterial pressure (*P* < 0.05) in nonhypertensive participants (*n* = 1 822) but not in hypertensive participants (*n* = 1 246). Furthermore, a high frequency of yogurt intake prevented hypertension; however, no additional antihypertensive effects were observed in patients already diagnosed with hypertension.

## 1. Introduction

Cardiovascular diseases (CVDs) are the leading cause of death worldwide, and high blood pressure is the leading modifiable risk factor for CVD [1]. One of the first clinical trials to show an inverse association between dairy intake and hypertension risk was the Dietary Approaches to Stop Hypertension (DASH) study, which involved a dietary pattern rich in fruits, vegetables, and low-fat dairy products, moderate in meat, fish, poultry, nuts, and beans, and low in sugar-sweetened beverages, sweets, and red meat [2]. Previous studies have reported that higher dairy consumption was associated with a decreased risk of cardiovascular comorbidities such as dyslipidemia, hypertension, insulin resistance, and type 2 diabetes [3, 4]. The mechanism behind the protective effects of yogurt on blood pressure is multifactorial. Yogurt is a form of dairy with a high concentration of casein, whey proteins, calcium, magnesium, and potassium. All of these have been linked to blood pressure lowering effects in animal studies, as well as some observational and experimental human studies [5]. In early clinical trials, the blood pressure lowering effect of dairy was largely attributed to its calcium content. However, others have shown that factors other than dietary calcium in dairy products may explain the beneficial effects on blood pressure and CVD risk [6]. Yogurt is made through fermentation, in which biologically active peptides such as isoleucine-proline-proline and valine-proline-proline are formed when milk proteins are catalyzed by proteolytic lactic acid bacteria such as *Lactobacillus helveticus*. Interestingly, the results of the few available longitudinal studies examining the direct effects of yogurt intake on blood pressure have not been conclusive [5, 7–9].

We designed a study to determine the effect of yogurt intake on blood pressure in people with different blood pressure ranges. One previous study indicated that higher yogurt intake in combination with an overall heart-healthy diet, as measured by the DASH diet score, was associated with a greater reduction in CVD risk among hypertensive men and women [10]. We aimed to either replicate or refute these initial findings of the potential benefits of habitual yogurt intake by examining the association between yogurt intake and blood pressure in hypertensive and nonhypertensive individuals.

## 2. Materials and Methods

### 2.1. Study Design and Settings

This study employed a cross-sectional analysis utilizing data from the National Health and Nutrition Examination Survey (NHANES). NHANES dietary data are used to describe the intake of foods, nutrients, food groups, and dietary patterns in the US population and in large sociodemographic groups to aid in the planning and evaluation of nutrition programs and policies. Usual dietary intake distributions can be estimated after adjusting for day-to-day variation. NHANES was approved by the NHANES Institutional Review Board (IRB) and the NCHS Research Ethics Review Board (ERB) (after 2003).

## 3. Participants and Data Collection

**Figure 1:**
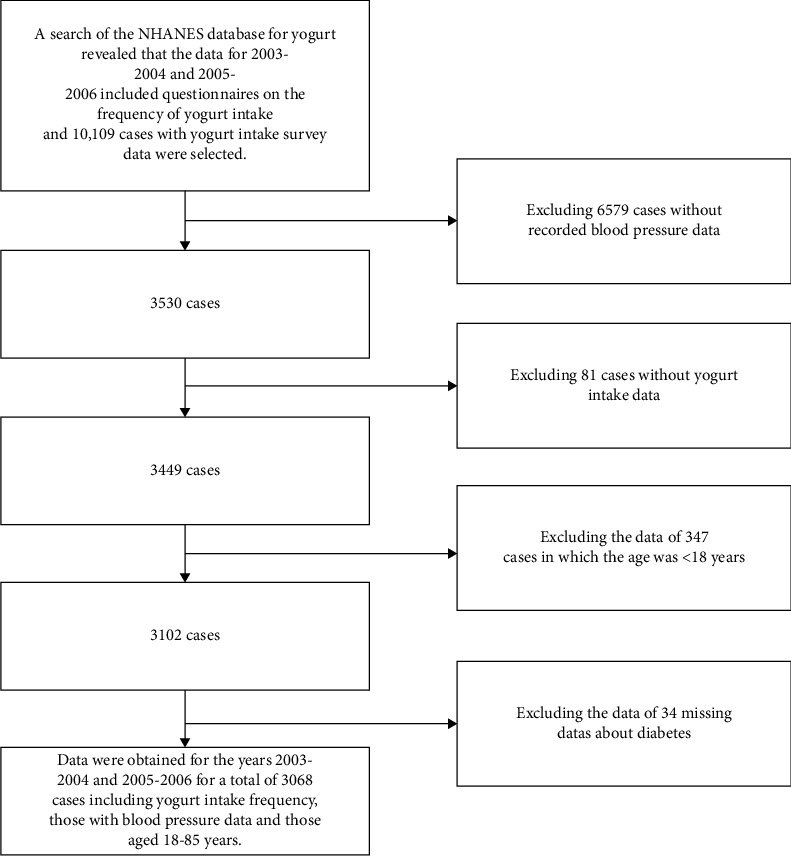
Flowchart of participants selection.

Our study included adults aged 18–85 years who participated in NHANES during the periods 2003–2004 and 2005–2006. Exclusion criteria encompassed NHANES participants lacking recorded blood pressure and yogurt intake data. The study population's flowchart is illustrated in Figure 1. NHANES data collection involves an in-home interview to gather demographic and basic health information, followed by a health examination in a mobile examination center (MEC). Trained interviewers, having completed an intensive training course facilitated by the US Department of Agriculture and the US Department of Health and Human Services, conducted the data collection.

### 3.1. Assessment of Yogurt Intake

Data were collected using a food frequency questionnaire (FFQ) which asked participants to indicate how often they consumed specific foods, including fruits, vegetables, meat, fish, eggs, bread, cereals, rice and pasta, legumes, dairy products, nuts, other snack foods, and beverages including alcohol. For each food, participants answered whether their habitual consumption was 1–6 times per year, 7–11 times per year, one time per month, 2–3 times per month, one time per month per week, two times per week, 3–4 times per week, 5–6 times per week, one time per day, or two times per day and above. We classified those who drank yogurt less than 7–11 times per year as the low-frequency group, and those who drank yogurt one time per month to one time per week and two times per week to two times per day and above as the high-frequency group. The yogurt intake frequency items were used to determine yogurt intake habits.

### 3.2. Blood Pressure Measurement

The primary study outcome was blood pressure parameters. NHANES BP examiners were certified in blood pressure measurement through the Shared Care Research and Education Consulting training. Three to four blood pressure measurements (systolic and diastolic) were performed at the MEC after 5 min of quiet rest in a seated position and determination of the maximum inflation level (MIL). Hypertension was defined as a systolic blood pressure (SBP) of ≥140 mmHg or a diastolic blood pressure (DBP) of ≥90 mmHg.

### 3.3. Covariate Assessment

A comprehensive exploration of potential confounding variables was conducted, encompassing sociodemographic factors, family history, and various diet and lifestyle factors. The selection of covariates was guided by insights from The Nurses' Health Study investigation [10] and previous analyses from the MSLS [11]. Final covariates included the following variables: age, marriage, income, diabetes, body mass index (BMI), high‐density lipoprotein cholesterol (HDL-C), and triglyceride (TG).

### 3.4. Statistical Analysis

Statistical analysis considered the complex survey design of the NHANES dataset. Data were classified as continuous variables and categorical variables. Continuous variables were further divided into two types based on the normality of their distribution. Normally distributed continuous variables are expressed as mean ± standard deviation, and comparisons between the two groups were carried out using Student's *t*-test. Non-normally distributed variables are expressed as interquartile range (IQR) ± median and comparisons between the two groups were conducted using the Wilcoxon rank-sum test. Categorical variables are expressed as percentages and were compared using the chi-square test. To assess the association between yogurt intake and blood pressure, we used unadjusted and adjusted multivariate linear regression models. After data processing, there were missing data for the following: BMI (*n* = 42), marriage (*n* = 1), income (*n* = 159), glycosylated hemoglobin (*n* = 235), total cholesterol (TC) (*n* = 111), TG (*n* = 1 655), low‐density lipoprotein cholesterol (LDL-C) (*n* = 1 688), and HDL-C (*n* = 111). We used multiple imputations based on five replications and a chained equation approach method via the mice package in *R* to maximize statistical power and minimize bias due to missing data. We also performed sensitivity analyses using a complete case analysis and repeated all analyses with the complete data cohort for comparison. Covariates were organized into three sets (models) as follows: model 1: no covariates were included; model 2: model 1 + age, marriage, and income; and model 3: model 2 + diabetes, BMI, HDL-C, and TG. The same models were used to examine blood pressure in hypertensive and nonhypertensive participants separately. We analyzed the hypertensive population into subgroups based on the use of antihypertensive medication or not. All of the analyses were performed with the statistical software packages R version 3.3.2 (http://www.R-project.org, The R Foundation) and Free Statistics software version 1.7.1. *P* values less than 0.05 (two-sided) were considered statistically significant.

### 3.5. Sensitivity Analysis

Sensitivity analysis was performed using the new definition of blood pressure. The American Heart Association recently updated the criterion for hypertension to SBP ≥130 mmHg or DBP ≥80 mmHg [12]. For primary analyses, we retained the previous definition of hypertension (140/90 mmHg) to reflect the long-standing and traditional definition of hypertension.

## 4. Results

### 4.1. Participants

We recruited a total of 10, 109 participants for our study from the NHANES database for the years 2003-2004 and 2005-2006. Initially, we excluded 6, 579 participants who did not have recorded blood pressure data. Afterward, we excluded an additional 81 participants who did not have data on yogurt intake. Then, we excluded 347 participants who were younger than 18 years old. Finally, we excluded 34 participants who did not have data on the history of diabetes mellitus. As a result, our final analysis included a total of 3, 068 participants.

### 4.2. Baseline Characteristics Based on Yogurt Intake

Baseline population characteristics based on yogurt intake are described in Table 1. More than one-third of participants reported never consuming yogurt (38.8%) and the rest of the participants reported consuming yogurt 1–6 times per year (14.9%), 7–11 times per year (9.0%), one time per month (7.1%), 2–3 times per month (10.8%), one time per week per month (3.5%), two times per week (5.9%), 3–4 times per week (5.8%), 5–6 times per week (1.9%), one time per day (1.9%), or two or more times per day (0.4%). Yogurt intake levels were combined into two categories. We classified those who drank yogurt less than 7–11 times per year as the low-frequency group and those who drank yogurt one time per month to one time per week and two times per week to two times per day and above as the high-frequency group. Participants in the group exposed to high frequency yogurt intake were more likely to be female, younger, and to have a higher income level.

### 4.3. Total Sample

The upper segment of Table 2 illustrates the association between yogurt intake frequency and blood pressure variables for the total sample. Multiple linear regression analysis results indicated significant inverse linear trends for SBP (*β* = −1.46; 95% CI = −2.72 and −0.2; *P* < 0.05), DBP (*β* = −1.59; 95% CI = −2.45 and −0.73; *P* < 0.05), and MAP (*β* = −2.08; 95% CI = −3.17 and −0.9; *P* < 0.05) after adjusting for all covariates (model 3). However, no significant linear trend was observed for pulse pressure (*β* = 0.13; 95% CI = −1.09 and 1.36; *P* > 0.05).

### 4.4. Hypertensive Individuals and Nonhypertensive Individuals

The lower part of Table 2 illustrates the association between yogurt intake frequency and blood pressure variables among hypertensive and nonhypertensive individuals. There were no significant associations between yogurt intake and SBP, DBP, MAP, or pulse pressure before and after adjustment for covariates among hypertensive patients (*P* > 0.05). Conversely, in nonhypertensive individuals, after controlling for all covariates (model 3), significant inverse linear trends were observed for SBP (*β* = −1.12; CI = −2.18 and −0.23; *P*=0.016), DBP (*β* = −1.45; CI = −2.38 and −0.52; *P*=0.002), and MAP (*β* = −1.85; CI = −2.93 and −0.78; *P*=0.001), but not for pulse pressure (*β* = 0.25; CI = −0.87 and 1.38; *P*=0.661).

### 4.5. Subgroup Analysis of the Hypertensive Group According to Antihypertensive Medication Uses

To analyze the potential masking effect of antihypertensive medication use on the relationship between yogurt intake and blood pressure,We divided the hypertensive individuals into subgroups for analyses based on the use of antihypertensive drugs or not.  Table 3 describes the relationship between the frequency of yogurt intake and blood pressure variables in the hypertensive and nonhypertensive medication groups. After adjusting for covariates, the results showed that yogurt consumption did not significantly affect SBP in individuals taking antihypertensive medication (*P* = 0.289) or in those not on such medication (*P* = 0.069). Similarly, there was no significant impact on DBP in the medicated group (*P* = 0.473) or in the nonmedicated group (*P* = 0.673). Furthermore, MAP and pulse pressure did not show significant changes attributable to yogurt intake in either group (*P* > 0.05). These outcomes align with the patterns noted in the hypertensive segment of our study population.

### 4.6. Sensitivity Analysis

**Figure 2:**
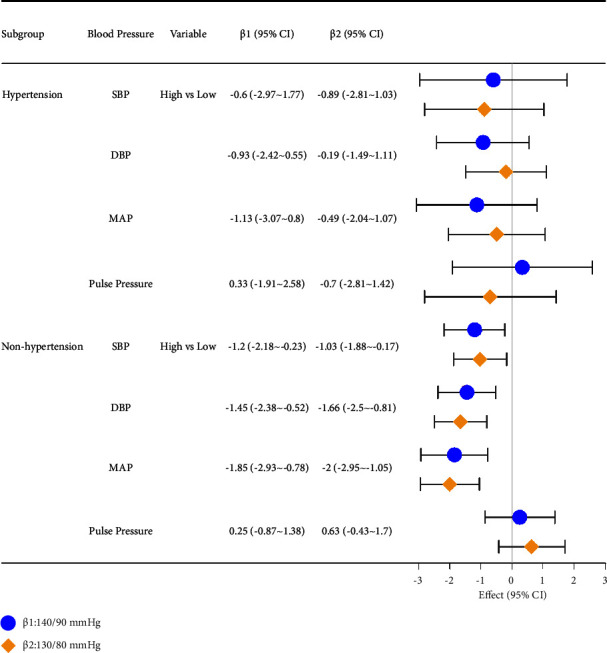
Different associations between yogurt intake frequency and blood pressure variables in hypertensive and nonhypertensive individuals after controlling for all covariates (*β*1: SBP ≥140 mmHg or DBP ≥90 mmHg and *β*2: SBP ≥130 mmHg or DBP ≥80 mmHg).

The sensitivity analysis based on the updated definition of hypertension (SBP ≥ 130 mmHg or DBP ≥ 80 mmHg) revealed essentially the same pattern of results as in the primary analysis (Figure 2). Significant inverse associations were observed for SBP, DBP, and MAP in the sample of persons without hypertension (defined as SBP ≥ 130 mmHg or DBP ≥80 mmHg). In the sample of hypertensive patients defined as SBP ≥130 mmHg or DBP ≥80 mmHg, no significant correlation was observed between yogurt intake and SBP, DBP, MAP, or pulse pressure before and after adjustment for covariates (*P* > 0.05).

## 5. Discussion

The current study examined the association between yogurt intake, blood pressure, and cardiovascular risk factors commonly associated with hypertension [13]. Our study employed multiple-factor regression analysis to explore the connection between yogurt intake and blood pressure in a large population. The results indicated that a higher frequency of yogurt consumption was linked to lower systolic blood pressure, diastolic blood pressure, and mean arterial pressure in the overall population. However, subgroup analysis revealed that this effect was not significant in individuals with hypertension, and we also found that the use of antihypertensive medications did not potentially affect this outcome. Conversely, in individuals without hypertension, increased yogurt intake was associated with a reduction of 1-2 mmHg in systolic blood pressure. Based on these findings, we suggest that yogurt consumption may have a preventive effect on the development of high blood pressure among the general population. The sensitivity analysis revealed essentially the same pattern of results as in the primary analysis.

Several previous observational studies have reported a positive association between yogurt consumption and improved blood pressure control in individuals with hypertension. For instance, a study conducted by Buendia JR et al. reported that higher yogurt intake was inversely associated with a reduced risk of CVD in hypertensive individuals [10, 14]. Scientific evidence has emerged supporting a beneficial relationship between the consumption of dairy products and the control of blood pressure in adults, for example, DASH notes the effects of dietary patterns on blood pressure [2].

However, our findings suggest that yogurt consumption does not have a significant antihypertensive effect in hypertensive patients. In a large prospective cohort of French women, overall consumption of dairy products was not associated with the risk of hypertension [15]. Another study showed that probiotics such as *Lactobacillus acidophilus* LA5 and *Bifidobacterium animalis* subsp. lactis BB-12 did not improve cardiovascular risk factors in hypertensive individuals [16]. We observed a significant association between increased yogurt intake frequency and lower blood pressure in individuals without hypertension. This finding implies that yogurt consumption may have a preventive effect on the development of high blood pressure among the general population.

Inconsistencies in results may stem from variations in milk product fat compositions and differences in study design, such as trial duration and statistical power [17–19]. While our research established the lack of potential impact of antihypertensive medication on the outcome, individuals with hypertension could be influenced by additional factors such as genetics, lifestyle, and other variables. Thus, the relationship between processed dairy products and blood pressure warrants further confirmation. In addition, fewer studies have explored the effects of yogurt or dairy products on blood pressure in nonhypertensive individuals.

Our findings provide new evidence demonstrating that the intake frequency of yogurt is related to positive blood pressure outcomes for nonhypertensive individuals, but not for hypertensive individuals. In our study, we did not find evidence of an association between yogurt consumption and blood pressure in hypertensive individuals. Some previous studies have reported results similar to ours [15]; that is, no association between overall fermented dairy or any specific type of fermented dairy product and hypertension risk was identified [20]. In our study, the intake frequency of yogurt was related to positive blood pressure outcomes for nonhypertensive individuals. In the CARDIA study, increased consumption of milk and dairy desserts was associated with a lower risk of hypertension [21], reinforcing our suggestion that yogurt consumption may have a preventive effect on high blood pressure development in the general population.

The precise mechanisms through which yogurt affect blood pressure remain unclear. Nonetheless, several hypotheses may explain why yogurt influences blood pressure levels in nonhypertensive populations but lacks a significant effect in hypertensive populations.

Calcium content: yogurt is a calcium-rich food, and calcium plays an important role in maintaining normal blood pressure levels [22]. Studies have shown that adequate calcium intake can help lower blood pressure .The addition of yogurt to the diet may have a positive effect on people with nonhypertension, as their blood pressure levels may be within the normal range, and calcium intake can help maintain normal blood pressure levels [23, 24]. However, in people who already have hypertension, calcium intake may have a more limited effect on blood pressure regulation.

Probiotics and bioactive peptides: yogurt is rich in probiotics, such as lactobacilli. These probiotics may affect blood pressure through several mechanisms. First, probiotics may improve the balance of intestinal flora and reduce the growth of harmful bacteria, thus helping to lower blood pressure [25, 26]. Second, the fermentation of lactic acid bacteria in yogurt produces bioactive peptides that have vasodilatory and antihypertensive effects, further assisting in lowering blood pressure [27–29]. The effects of these probiotics and bioactive peptides may be more pronounced in nonhypertensive individuals, as they may have more normal blood pressure levels and may be more sensitive to these active ingredients. Hypertension is a complex disease influenced by multiple factors [30]. People with hypertension may be influenced by other factors, such as genetic factors, lifestyle, and medication, which may mask the potential effect of yogurt on blood pressure [30]. Different individuals may respond differently to the ingredients and probiotics in yogurt. In addition, the hypertensive population already has elevated baseline blood pressure and may require more potent interventions to significantly alter blood pressure levels. It is important to note that the abovementioned hypotheses are speculative, and further studies are needed to validate and deepen our understanding. Due to variations in the effects of yogurt on blood pressure in different populations, future studies should explore specific biological mechanisms and investigate individual differences in greater depth. This will provide more accurate guidance for dietary interventions and management of cardiovascular disease.

Despite its contributions, our study has several limitations. First, one significant limitation of our study is its cross-sectional design, which restricts our ability to establish causality. Cross-sectional studies are observational in nature and capture data at a single point in time. As a result, they can only determine associations or correlations between variables, rather than establishing a cause-and-effect relationship. In our study, we assessed the relationship between yogurt intake and blood pressure measures, but we cannot determine whether yogurt consumption directly affects blood pressure or if other factors are involved. Longitudinal studies that follow individuals over time would be necessary to investigate the temporal sequence of events and provide stronger evidence for causal relationships. Second, yogurt intake levels, as captured by the FFQ, are subject to some measurement error. The FFQ does not account for serving size variations between individuals, and participants were not required to specify the fat content or sugar content of the consumed yogurt, limiting additional analyses. However, the study's robustness is enhanced by its large sample size, providing relatively reliable results.

Our study provided valuable insights into the association between yogurt and blood pressure outcomes in nonhypertensive individuals. In SBP model 3, a high frequency of yogurt intake was correlated with a reduction in SBP of 1.2 mmHg. Although this is a relatively small change, a change in blood pressure of this magnitude could lead to significant differences at the population level. Indeed, a meta-analysis examining the relationship between blood pressure and vascular mortality reported that a reduction of 2 mmHg in SBP translates to a 10% lower risk of stroke and cardiovascular mortality [31]. For nonhypertensive people, reducing blood pressure by increasing the intake frequency of yogurt is not the ultimate goal, and this group of people should instead adopt a healthy lifestyle to reduce the incidence of CVD events.

## 6. Conclusions

Upon accounting for demographic, lifestyle, and cardiovascular variables, our findings suggest that augmenting the frequency of yogurt intake has the potential to lower blood pressure in individuals without hypertension, but not in those already diagnosed with hypertension. Thus, yogurt has emerged as a promising food for the prevention of hypertension.

## Figures and Tables

**Table 1:** Demographic and health characteristics for the total sample (*n* = 3 068) according to the level of yogurt intake frequency.

Variables	Total (*n* = 3068)	Low-frequency group (*n* = 1648)	High-frequency group (*n* = 1420)	*P* value
*Gender*				<0.001
Male	1429 (46.6)	944 (57.3)	485 (34.2)	
Female	1639 (53.4)	704 (42.7)	935 (65.8)	
Age	46.6 ± 19.5	48.8 ± 19.5	44.0 ± 19.2	<0.001
BMI (kg/m^2^)	28.3 ± 6.1	28.4 ± 6.1	28.1 ± 6.2	0.164
*Marriage*				0.802
Married	1772 (57.8)	955 (58)	817 (57.5)	
Widow	1295 (42.2)	692 (42)	603 (42.5)	
*Diabetes*				<0.001
Yes	291 (9.5)	185 (11.2)	106 (7.5)	
No	2777 (90.5)	1463 (88.8)	1314 (92.5)	
Glycosylated hemoglobin (g/dl)	0.5 ± 0.2	0.6 ± 0.2	0.5 ± 0.1	0.027
*Income*				<0.001
Low	859 (29.5)	475 (30.3)	384 (28.7)	
Middle	1115 (38.3)	644 (41)	471 (35.2)	
High	935 (32.1)	451 (28.1)	484 (36.1)	
TC (mmol/L)	5.2 ± 1.1	5.2 ± 1.2	5.2 ± 1.1	0.447
TG (mmol/L)	1.4 (0.9, 2.0)	1.4 (1.0, 2.0)	1.3 (0.9, 2.0)	0.028
LDL-C (mmol/L)	2.9 (2.3, 3.5)	2.9 (2.4, 3.6)	2.9 (2.3, 3.5)	0.089
HDL-C (mmol/L)	1.3 (1.1, 1.6)	1.3 (1.1, 1.6)	1.4 (1.1, 1.7)	<0.001
SBP (mmHg)	125.0 ± 21.2	127.1 ± 21.5	122.5 ± 20.6	<0.001
DBP (mmHg)	70.1 ± 12.3	71.0 ± 12.4	69.0 ± 12.2	<0.001
MAP (mmHg)	111.7 ± 16.5	113.4 ± 16.4	109.8 ± 16.3	<0.001
Pulse pressure (mmHg)	54.9 ± 19.9	56.1 ± 20.6	53.4 ± 19.0	<0.001

BMI, body mass index; TC, total cholesterol; TG, triglyceride; HDL-C, high‐density lipoprotein cholesterol; LDL-C, low‐density lipoprotein cholesterol; SBP, systolic blood pressure; DBP, diastolic blood pressure; MAP, mean arterial pressure. Income categories based on the profitability index ratio (PIR) values low (PIR ≤1.3); middle (1.3 < PIR < 3.5); high (PIR ≥ 3.5).

**Table 2:** Cross-sectional associations between yogurt and blood pressure in a sample of the total sample (*n* = 3 068), persons (*n* = 1 246) with hypertension, and persons (*n* = 1 822) with normal blood pressure.

		Variable	Model 1		Model 2		Model 3	
			*β* 95% CI	*P* value	*β* 95% CI	*P* value	*β* 95% CI	*P* value
Total sample	SBP	Yogurt intake frequency (high vs low)	−4.65 (−6.15∼−3.15)	<0.001	−1.59 (−2.85∼−0.32)	0.014	−1.46 (−2.72∼−0.2)	0.023
	DBP		−1.98 (−2.85∼−1.11)	<0.001	−1.7 (−2.56∼−0.83)	<0.001	−1.59 (−2.45∼−0.73)	<0.001
	MAP		−3.53 (−4.69∼−2.37)	<0.001	−2.22 (−3.33∼−1.12)	<0.001	−2.08 (−3.17∼−0.99)	<0.001
	Pulse pressure		−2.67 (−4.08∼−1.26)	<0.001	0.11 (−1.11∼1.33)	0.860	0.13 (−1.09∼1.36)	0.833
Hypertension	SBP		−1.25 (−3.79∼1.29)	0.334	−0.33 (−2.7∼2.04)	0.783	−0.6 (−2.97∼1.77)	0.619
	DBP		−0.38 (−1.93∼1.17)	0.633	−0.81 (−2.3∼0.69)	0.289	−0.93 (−2.42∼0.55)	0.218
	MAP		−0.8 (−2.74∼1.15)	0.423	−0.92 (−2.87∼1.03)	0.356	−1.13 (−3.07∼0.8)	0.252
	Pulse pressure		−0.87 (−3.49∼1.75)	0.514	0.48 (−1.77∼2.72)	0.678	0.33 (−1.91∼2.58)	0.772
Nonhypertension	SBP		−2.01 (−3.05∼−0.97)	<0.001	−1.42 (−2.41∼−0.42)	0.005	−1.2 (−2.18∼−0.23)	0.016
	DBP		−1.73 (−2.68∼−0.78)	<0.001	−1.58 (−2.52∼−0.65)	0.001	−1.45 (−2.38∼−0.52)	0.002
	MAP		−2.4 (−3.53∼−1.28)	<0.001	−2.06 (−3.14∼−0.97)	<0.001	−1.85 (−2.93∼−0.78)	0.001
	Pulse pressure		−0.28 (−1.4∼0.84)	0.625	0.17 (−0.96∼1.29)	0.770	0.25 (−0.87∼1.38)	0.661

Model 1, no covariates were included; model 2, model 1 + age, marriage, and income; model 3, model 2 + diabetes, BMI, HDL-C, and TG.

**Table 3:** Subgroup analysis of the hypertensive group according to antihypertensive medication use.

	Subgroup	Variable	Model 1		Model 2		Model 3	
			*β* 95% CI	*P* value	*β* 95% CI	*P* value	*β* 95% CI	*P* value
SBP	Medicated group	Yogurt intake frequency (high vs low)	−1.38 (−4.75∼1.98)	0.421	−1.4 (−4.64∼1.83)	0.395	−1.75 (−5∼1.49)	0.289
	Nonmedicated group		−5.69 (−11.33∼−0.05)	0.049	−4.38 (−9.36∼0.59)	0.086	−4.68 (−9.7∼0.34)	0.069
DBP	Medicated group		−0.48 (−2.46∼1.51)	0.640	−0.46 (−2.32∼1.4)	0.625	−0.69 (−2.56∼1.19)	0.473
	Nonmedicated group		−1.03 (−4.37∼2.32)	0.549	−0.08 (−3.43∼3.28)	0.965	−0.72 (−4.05∼2.61)	0.673
MAP	Medicated group		−0.94 (−3.46∼1.59)	0.468	−0.93 (−3.43∼1.57)	0.465	−1.27 (−3.78∼1.24)	0.321
	Nonmedicated group		−2.92 (−7.45∼1.61)	0.208	−1.54 (−5.93∼2.86)	0.494	−2.28 (−6.64∼2.08)	0.307
Pulse pressure	Medicated group		−0.91 (−4.34∼2.53)	0.604	−0.94 (−3.95∼2.07)	0.540	−1.07 (−4.09∼1.96)	0.489
	Nonmedicated group		−4.67 (−9.72∼0.39)	0.072	−4.31 (−8.79∼0.17)	0.061	−3.96 (−8.55∼0.62)	0.092

Model 1, no covariates were included; model 2, model 1 + age, marriage, and income; model 3, model 2 + diabetes, BMI, HDL-C, and TG.

## Data Availability

Publicly available datasets were analyzed in this study. These data can be found in NHANES Questionnaires, Datasets, and Related Documentation (https://cdc.gov).
